# Circadian immunoregulation in cutaneous wound healing: from immune cell temporal heterogeneity to chronotherapeutic strategies

**DOI:** 10.3389/fimmu.2026.1917390

**Published:** 2026-08-26

**Authors:** Ziyi Luo, Junzhe Chen, Shaoxiang Yuan, Yuezhong Chen, Jiaqi Liu, Jingbo Hou, Feiyang Linghu, Chengliang Deng, Shune Xiao

**Affiliations:** 1 Department of Burns and Plastic Surgery, Affiliated Hospital of Zunyi Medical University, Zunyi, Guizhou, China; 2 Collaborative Innovation Center of Tissue Repair and Regenerative Medicine, Zunyi, Guizhou, China

**Keywords:** chronic wounds, chronotherapy, circadian rhythm, cutaneous wound healing, immune regulation, macrophage activation states, neutrophils

## Abstract

Cutaneous wound healing is not only a spatially coordinated reparative process but also a temporally organised immune programme shaped by circadian rhythms. This Review summarises how the central clock, peripheral clocks and core clock genes regulate time-dependent processes during skin repair, including immune cell recruitment, activation, resolution of inflammation and tissue remodelling. We focus on circadian regulation of neutrophil trafficking and effector functions, together with macrophage temporal heterogeneity and state transitions during inflammatory resolution. We further discuss the potential contributions of dendritic cells, mast cells, innate lymphoid cells and T cells to the temporal regulation of wound immunity. Circadian disruption associated with diabetes, ageing, sleep disturbance, obesity and aberrant light exposure may desynchronise immune responses, thereby promoting persistent inflammation, increased oxidative stress, heightened protease activity and enhanced formation of neutrophil extracellular traps, ultimately contributing to the development and progression of chronic non-healing wounds. Finally, we discuss chronotherapeutic strategies that align wound interventions, metabolic regulation, light exposure, sleep and feeding time with endogenous circadian rhythms. Incorporating circadian timing into wound-healing research may help redefine the temporal heterogeneity of immune cells and open new avenues for circadian-guided precision therapy for refractory chronic wounds.

## 1 Introduction

The skin, as the largest organ of the human body, provides an extensive interface with the external environment and plays a central role in maintaining barrier integrity and initiating immune defence against external challenges. Beyond its barrier function, the skin is essential for thermoregulation, sensory perception, immune surveillance and the preservation of tissue homeostasis (1). Upon injury induced by trauma, infection, ischaemia or metabolic disturbance, a highly coordinated reparative programme is initiated to restore tissue integrity. Skin wound repair is a highly coordinated biological programme that begins with haemostasis and inflammation, followed by proliferative tissue formation and matrix remodelling. Throughout this process, platelets, immune cells and stromal cells engage in dynamic crosstalk mediated by cytokines, chemokines, growth factors and extracellular matrix reorganisation (2). However, although this canonical framework captures the dynamic changes in cellular composition and the local microenvironment during repair, it has paid comparatively little attention to time as a fundamental regulatory axis (3, 4).

In recent years, advances in circadian biology have revealed that circadian rhythms have indispensable roles in a wide range of physiological processes. Beyond the regulation of sleep and endocrine function, circadian rhythms also participate in skin barrier homeostasis, cell migration, inflammatory responses and tissue regeneration (5). As a peripheral organ directly exposed to light, temperature fluctuations and other environmental cues, the skin possesses a prominent local clock system (6). Keratinocytes, fibroblasts, endothelial cells and immune cells are all regulated by peripheral clocks in this context. Amongst these cellular compartments, immune cells have a particularly important role, as immune responses represent a key bridge between circadian timing and wound repair. Whether an acute wound heals successfully depends, to a large extent, on whether inflammation is initiated, amplified, constrained and ultimately resolved at the appropriate time. Rather than acting as static participants in tissue repair, immune cells such as neutrophils, macrophages, dendritic cells, mast cells, innate lymphoid cells and T cells exhibit rhythmic recruitment, activation, metabolic reprogramming and functional transitions within defined temporal windows. When this rhythmic regulation is disrupted, early antimicrobial defence may be insufficient, whereas persistent retention of inflammatory cells, protease release, oxidative stress and mistimed reparative signals may drive the transition from acute inflammation to a chronic non-healing state. Although emerging studies have begun to investigate the relationship between cutaneous circadian rhythms and wound healing, systematic integration of wound-repair mechanisms from the perspective of immune-cell rhythmicity remains limited. Reconsidering wound healing through a temporal lens may help explain why acute wounds can progress in an orderly manner towards repair, whereas chronic wounds remain trapped in a state of persistent inflammation. This Review aims to summarise how circadian rhythms regulate immune-cell behaviour during cutaneous wound healing and to further discuss chronotherapeutic intervention strategies and their prospects for clinical translation.

## 2 Circadian rhythms in skin and wound repair

### 2.1 Central and peripheral clocks

Before considering how circadian rhythms influence skin biology and wound repair, it is first necessary to define the biological clock and the circadian rhythm.

Circadian rhythmicity is generated and sustained by an internal clock system that aligns organismal physiology with the daily alternation of light and darkness. This system operates with an approximately 24-hour periodicity and is present in nearly all unicellular and multicellular organisms. By aligning thermoregulation, cardiovascular activity, sleep and feeding behaviours, endocrine output, metabolic balance and immune responses with daily environmental cycles, the circadian system supports temporal adaptation and physiological homeostasis (7, 8). The mammalian circadian system consists of a central clock and numerous peripheral oscillators. The central clock, situated in the suprachiasmatic nucleus (SCN) of the hypothalamus, receives photic input from the retina and coordinates peripheral tissues by transmitting timing cues through neuronal, humoral and endocrine routes. Melatonin, glucocorticoids, fluctuations in body temperature and autonomic nervous signals are amongst the key mediators that synchronise peripheral clocks. Peripheral clocks are widely distributed in the skin, liver, adipose tissue, muscle, intestine and immune cells, and likewise have essential roles in maintaining rhythmic physiology (9). Peripheral clocks are regulated by the central clock, but they are also modulated by additional systemic and local timing cues, including feeding time, physical activity, temperature changes, inflammatory stimuli and tissue injury (10). As a genetically encoded timekeeping system, the biological clock coordinates transcriptional and signalling networks across tissues to maintain organismal homeostasis. Cellular rhythms are not governed solely by the central clock; when central clock control is weakened or absent, cells can also rely on autonomous clocks to respond to diverse stimuli. As a peripheral organ directly exposed to light, temperature, microorganisms and mechanical stress, the skin exhibits pronounced local rhythmicity. Keratinocytes, fibroblasts, endothelial cells and multiple immune-cell populations possess cell-autonomous clocks, which regulate cell migration, proliferation, inflammatory responses and tissue repair through rhythmic gene expression (7). Thus, during wound healing, the central clock provides systemic temporal cues, whereas local peripheral clocks in the skin further integrate signals from the injury microenvironment. Together, these timing systems shape the initiation of wound inflammation, its subsequent resolution and the process of tissue reconstruction.

### 2.2 Core clock genes and molecular feedback loops

Circadian rhythms are driven by an endogenous timekeeping system, the molecular basis of which is a transcriptional regulatory network composed of clock genes. This network generates approximately 24-hour oscillations through transcriptional–translational feedback loops (TTFLs), thereby regulating rhythmic changes in diverse physiological functions and behavioural activities (11). This process is governed by a conserved molecular clockwork composed of several core genes, including circadian locomotor output cycles kaput (CLOCK), brain and muscle ARNT-like protein 1 (BMAL1), cryptochromes (CRY1/2), period proteins (PER1–3), and nuclear receptor subfamily 1 group D members 1 and 2 (NR1D1/2, also known as REV-ERBα/β) (12). The circadian system operates through three interlinked TTFLs. During the day, BMAL1 and CLOCK form a specific heterodimer that binds to E-box elements in the promoters of target genes and drives the transcription of PER and CRY. After transcription, PER and CRY mRNAs are exported to the cytoplasm for translation. PER proteins interact with casein kinase 1δ/ϵ (CK1δ/ϵ) in the cytoplasm and gradually accumulate. At night, PER and CRY proteins form heterodimers and translocate into the nucleus, where they inhibit CLOCK–BMAL1 activity, thereby suppressing further transcription of PER and CRY genes. As PER and CRY proteins undergo sequential phosphorylation, ubiquitination and proteasomal degradation, CLOCK–BMAL1 activity is restored, initiating a subsequent transcriptional phase. This self-perpetuating oscillatory process underlies the canonical 24-hour negative feedback loop of the circadian clock (13, 14). In addition to the classical CLOCK–BMAL1–PER/CRY loop, circadian rhythms are regulated by auxiliary feedback loops. The second, RORE-mediated loop mainly controls BMAL1 expression: ROR family transcription factors promote BMAL1 transcription, whereas REV-ERBs repress it. Through this balance of positive and negative regulation, the RORE loop stabilises core clock oscillations (15). The third, D-box loop, consists of PAR-bZIP transcription factors, including DBP, TEF and HLF, together with the repressor E4BP4/NFIL3, and primarily links the core clock machinery to downstream rhythmic output genes (16). These nested feedback loops enable cells to translate circadian signals into rhythmic changes in gene expression, metabolic state and immune function. During cutaneous wound repair, core clock genes influence multiple cellular events, ranging from keratinocyte and fibroblast migration and proliferation to immune-cell trafficking, activation and cytokine release by neutrophils, macrophages, dendritic cells and T cells. Accordingly, abnormalities in core clock genes or disruption of circadian rhythmicity may lead to dysregulated wound inflammation, mistimed reparative signalling and, ultimately, impaired wound-healing quality (4).

### 2.3 Circadian regulation of skin-resident cells during wound healing

The skin is composed mainly of the epidermis, dermis and subcutaneous tissue, and functions as a barrier organ with pronounced peripheral clock properties (17). Keratinocytes constitute the major cellular component of the epidermis, which contains no blood vessels and depends primarily on dermal diffusion for nutritional support; it has central roles in barrier maintenance and re-epithelialisation (18). The dermis lies beneath the epidermis and is usually divided into the papillary and reticular layers. Rich in collagen fibres, blood vessels, immune cells and fibroblasts, it constitutes a core site for inflammatory responses, granulation tissue formation, angiogenesis and extracellular matrix remodelling (19). The subcutaneous tissue is located deep to the dermis and contains abundant subcutaneous fat, which primarily contributes to energy storage and thermal insulation (20). Similar to other organs and tissues, skin cells do not maintain their functions in a static state; rather, they are regulated by local clocks and display time-dependent changes in barrier function, cell migration, proliferation and immune responses (21).

Following injury caused by mechanical forces, microbial infection or other insults, the organism initiates a coordinated series of responses to promote wound healing. This process requires the integrated actions of multiple cell types, including keratinocytes, fibroblasts, immune cells such as neutrophils and macrophages, and endothelial cells, which cooperate to execute the haemostatic, inflammatory, proliferative and remodelling phases of repair (4, 22) In the early phase after injury, vasoconstriction, platelet aggregation and fibrin clot formation collectively achieve haemostasis (23). After tissue damage, neutrophils are amongst the earliest leukocytes to accumulate at the wound site, guided by damage-associated molecular patterns (DAMPs), hydrogen peroxide (H_2_O_2_), lipid mediators and chemokines released within the injured microenvironment. Their rapid influx supports initial pathogen control and the clearance of necrotic tissue. Subsequently, monocytes and macrophages are progressively recruited within 48–96 hours after injury, where they participate in inflammatory amplification, removal of necrotic debris and resolution of inflammation. As the inflammatory phase subsides, keratinocyte migration promotes re-epithelialisation, endothelial cells mediate neovascularisation, and fibroblasts enter and become activated within the wound, with a subset further differentiating into myofibroblasts to support granulation tissue formation and extracellular matrix deposition (24, 25). Finally, the wound enters the remodelling phase, during which newly formed epithelial tissue and scar tissue continue to mature (26). Notably, these cellular events are not driven passively by injury-derived signals alone. Keratinocytes, fibroblasts, endothelial cells and immune cells all possess cell-autonomous clocks; therefore, circadian disruption may interfere with wound healing by perturbing the temporally ordered functions of multiple skin-resident cell populations.

Existing studies suggest that the clocks of skin structural cells can directly influence the efficiency of wound repair. Bigliardi et al. (27) showed, using delta opioid receptor (DOPr) knockdown experiments, that keratinocytes lacking DOPr fail to maintain PER2 rhythmicity, suggesting that the keratinocyte clock may contribute to epidermal homeostasis and injury repair. Hoyle et al. (28) demonstrated that fibroblasts possess a cell-autonomous circadian regulatory mechanism, through which the endogenous clock modulates actin cytoskeletal dynamics, thereby influencing fibroblast migratory capacity and recruitment to the wound area. In the same study, Hoyle et al. further demonstrated that injuries occurring during the active phase were associated with more robust fibroblast infiltration and mobilisation, as well as faster wound closure, indicating that the rhythmic function of fibroblasts may represent an important mechanism linking peripheral clocks to the efficiency of skin wound repair. Another study similarly found that when scratch injury was performed in synchronised mouse skin fibroblast monolayers at the peak of PER2 expression, the wound was almost completely healed after 60 hours; by contrast, monolayers injured at the trough of PER2 expression remained far from healed. Nakazato et al. (29) further showed that the length of primary cilia in fibroblasts is regulated by clock genes. When this rhythm was pharmacologically disrupted, or when Bmal1 was knocked out, fibroblast migration was impaired, leading to delayed wound repair. These findings suggest that circadian disruption can interfere with wound healing by affecting fibroblast migration. Together, these studies indicate that the fibroblast clock may be one of the key mechanisms connecting peripheral circadian rhythms with the efficiency of wound closure. Beyond structural cells, skin immune cells are also regulated by circadian rhythms. Core clock genes impose rhythmic regulation on diverse skin-resident and infiltrating immune cells, encompassing Langerhans cells and γδ T cells in the epidermis, together with neutrophils, macrophages, mast cells, dendritic cells and T cells in the dermis. For example, CLOCK can participate in the progression of inflammatory skin disease by regulating IL-23R expression in γ δ^+^ T cells (30), whereas BMAL1 influences antiviral gene expression in Langerhans cells (31). In addition, macrophage cytokine production and phagocytosis, mast-cell IgE-mediated degranulation, and the recruitment of neutrophils and macrophages to sites of skin inflammation all display circadian rhythmicity, with some of these rhythms being abolished when clock function is lost (32–35). Thus, circadian regulation in cutaneous wound healing is not confined to a single cell type; rather, it operates through temporally coordinated interactions between structural cells and immune cells, collectively shaping inflammatory initiation, tissue reconstruction and repair outcomes.

**Figure 1:**
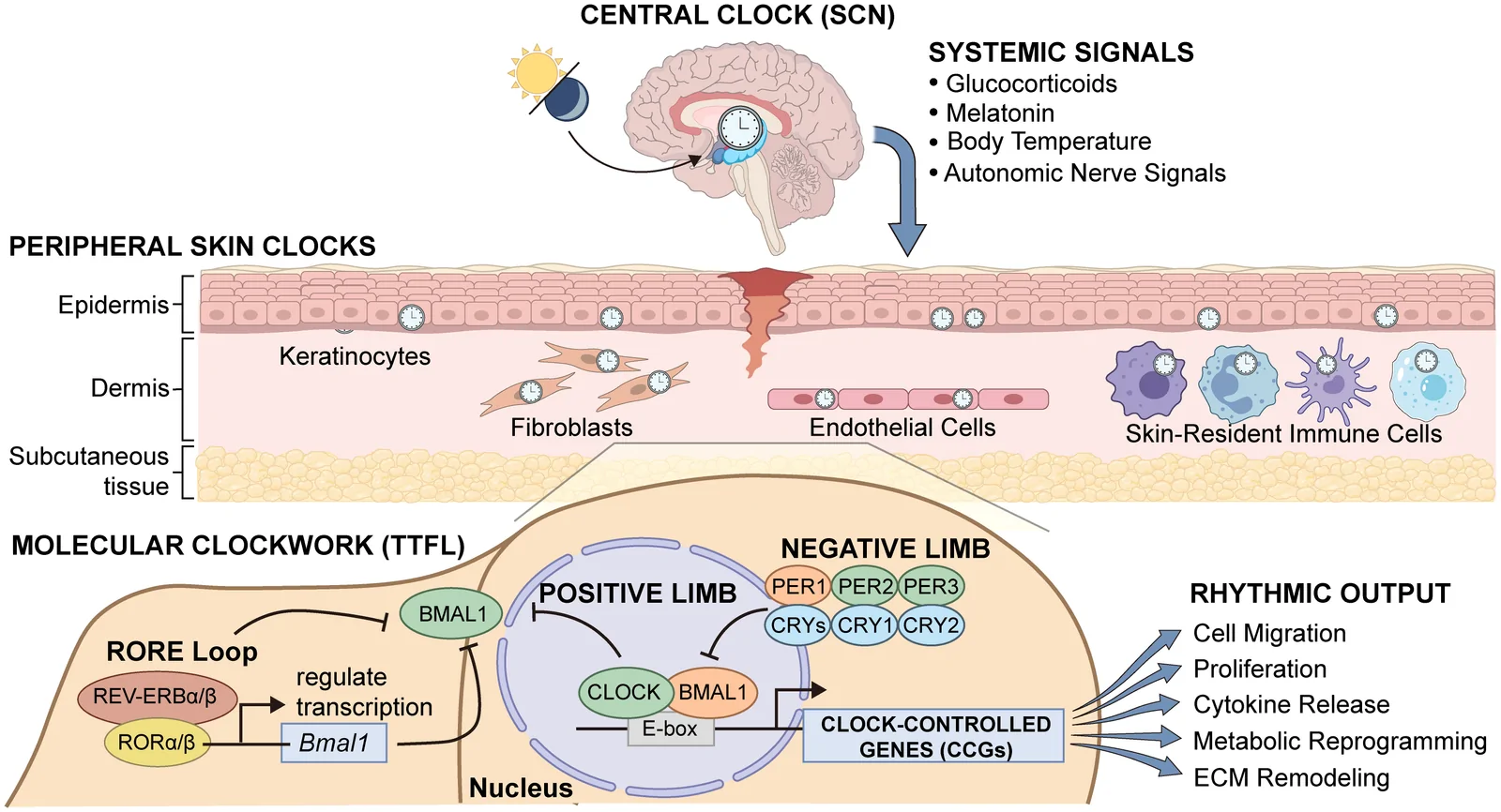
Hierarchical circadian regulation of skin wound repair. Skin wound repair is governed by a hierarchical circadian system consisting of a central pacemaker in the SCN and peripheral clocks in skin-resident cells. Signals such as glucocorticoids, melatonin, body temperature and autonomic nerve activity synchronise peripheral clocks in keratinocytes, fibroblasts, endothelial cells and skin-resident immune cells across the skin layers. At the molecular level, circadian timing is generated by the TTFL, in which CLOCK–BMAL1 drives expression of PER and CRY, which in turn repress CLOCK–BMAL1 activity, whilst ROR and REV-ERB regulate Bmal1 transcription. These oscillatory circuits control the rhythmic expression of clock-controlled genes, thereby influencing wound-healing processes such as cell migration, proliferation, cytokine production, metabolic reprogramming and extracellular matrix remodelling.

Together, these central, peripheral and molecular clock components provide a temporal framework for cutaneous wound repair (**Figure 1**).

### 2.4 Circadian regulation of dynamic immune-cell changes during wound healing

Cutaneous wound healing is a highly dynamic biological process with a defined temporal order, and its successful progression depends on the coordinated actions of immune cells, structural cells and vascular cells within specific temporal windows (36). In the early phase after injury, platelet aggregation and fibrin clot formation initiate haemostasis. Subsequently, innate immune cells, including neutrophils, monocytes and macrophages, rapidly infiltrate the wound, where they clear pathogens and necrotic tissue and establish the early inflammatory microenvironment (37). As inflammation becomes progressively constrained, keratinocyte migration, angiogenesis, fibroblast activation and granulation tissue formation collectively drive the wound into the proliferative phase (38). Finally, extracellular matrix reorganisation, collagen maturation and scar remodelling determine the quality of tissue repair (39). During this process, immune cells do not exert a single continuous function. Instead, they are sequentially recruited, activated, functionally reprogrammed and gradually withdrawn in accordance with the progression of wound healing. Therefore, the magnitude, duration and phase-specific transition of immune responses are key determinants of whether an acute wound heals successfully. Circadian rhythms have indispensable roles in this temporally ordered emergence, function and resolution of immune-cell activity. Indeed, early studies showed that cells of both the innate and adaptive immune systems exhibit circadian rhythmicity in gene expression (40). For example, Bree et al. (41) found that, in older adults, morning BCG vaccination induced a stronger trained immune response than evening vaccination, suggesting that the intensity of immune responses to stimulation can be shaped by time of day. Three circadian regulatory loops have been shown to control immune function through BMAL1, REV-ERB, PER and CRY genes. The BMAL1-centred loop determines when immune cells migrate and initiate inflammation; the REV-ERB loop constrains excessive amplification of inflammatory responses; and the PER/CRY loop establishes negative feedback by inhibiting CLOCK–BMAL1 activity. Together, these interconnected circuits maintain the rhythmicity, controllability and homeostasis of the immune system along the temporal axis.

Thus, the local immune response within a wound can be understood as a temporally organised process jointly regulated by circadian rhythms and the injury microenvironment. On the one hand, endogenous circadian rhythms influence immune-cell distribution in the circulation, tissue recruitment and inflammatory mediator release. On the other hand, DAMPs, chemokines, growth factors, metabolic cues and hypoxic signals released locally after injury further reshape immune-cell states, enabling a gradual transition from early host defence to inflammatory resolution, tissue reconstruction and matrix remodelling. In this process, neutrophils, macrophages, mast cells, dendritic cells and T cells do not act in isolation; instead, they form interconnected immune networks across distinct stages of repair (42). If this rhythmic regulation becomes imbalanced, early antimicrobial and clearance responses may be insufficient, whereas persistent inflammation, protease release, oxidative stress and mistimed reparative signalling may promote chronic wounds, fibrosis or pathological scarring (43). Therefore, understanding the dynamic changes of immune cells during wound healing from a circadian perspective may help reveal the key mechanisms that govern the transition from acute inflammation to tissue repair and provide a theoretical basis for chronotherapeutic strategies for chronic non-healing wounds.

## 3 The innate immune system and circadian rhythms

### 3.1 Neutrophil rhythms and acute wound inflammation

As the most abundant innate immune cells, neutrophils are highly responsive to injury signals and represent one of the earliest immune cell populations recruited to cutaneous wounds. Following tissue damage, platelet aggregation, complement activation, DAMPs released by necrotic cells, and chemokines produced by local epithelial cells, endothelial cells and stromal cells together establish an inflammatory gradient that recruits neutrophils to the site of injury within hours. Neutrophil recruitment involves a coordinated sequence of rolling, adhesion, crawling and transendothelial migration (44). At the site of injury, neutrophils mediate host defence and clearance of damaged tissue by engaging multiple effector programmes, including phagocytosis, degranulation, reactive oxygen species production, MMP-8 release and the generation of neutrophil extracellular traps (NETs). In doing so, they establish an early inflammatory microenvironment that supports subsequent monocyte and macrophage recruitment, angiogenesis, cell migration and matrix remodelling (45).

Owing to their short lifespan, neutrophils were long regarded as relatively homogeneous terminally differentiated cells. More recent work, however, has shown that neutrophils are dynamic immune cells with pronounced functional heterogeneity and circadian features in circulation, tissue distribution, ageing, homing and inflammatory responses. Neutrophil release from the bone marrow, circulation in peripheral blood, distribution within the marginal pool and homing to tissues are not constant across the day; rather, they are jointly regulated by the core clock machinery and chemokine axes (46–48) Studies have shown that BMAL1 can influence rhythmic neutrophil “ageing” by regulating CXCL2 expression. CXCR2 signalling promotes the ageing programme of circulating neutrophils, whereas CXCR4 is associated with their homing and clearance (32). Here, “ageing” does not simply refer to accelerated cell death. Instead, it may reflect an extension of cellular lifespan accompanied by time-dependent phenotypic changes, including alterations in adhesion molecules, chemokine receptors, inflammatory responsiveness and tissue-homing capacity. This implies that neutrophils entering the wound at different times of day may occupy distinct functional states and therefore exert different roles during wound healing.

The timing of injury may influence the number, maturation state and migratory capacity of neutrophils that can be mobilised by the host. If injury occurs when neutrophil mobilisation capacity is high, the wound may acquire antimicrobial protection more rapidly; if injury occurs when mobilisation capacity is low, or when neutrophils are in a less favourable functional state, early host defence may be weakened. In human burn datasets, Hoyle et al. found that burns sustained during the daytime healed approximately 60% faster than burns sustained at night (28), suggesting that the timing of injury can affect repair efficiency. Although this phenomenon cannot be attributed entirely to neutrophils, neutrophil rhythmicity, as a central effector of acute inflammation, is likely to represent an important immunological link between injury timing and differences in early inflammatory responses. Second, neutrophil rhythms may shape the protease–matrix balance within the wound microenvironment. Under physiological conditions, neutrophil-derived proteases help remove necrotic tissue and participate in early matrix remodelling. However, when circadian disruption leads to persistent neutrophil infiltration or impaired clearance, protease activity may shift from a “remodelling tool” to a “tissue-damaging factor”. This can result in degradation of collagen, fibronectin, basement membrane structures and growth factors, thereby impairing the migration and proliferation of fibroblasts and keratinocytes. Persistent inflammation, a high-protease environment and neutrophil retention, which are commonly observed in chronic wounds, may reflect this imbalance. Studies of NETs further suggest that excessive NET formation can amplify inflammatory responses and impair angiogenesis, thereby delaying wound healing (49). In addition, loss of neutrophil rhythmicity may drive the transition from acute inflammation to chronic non-healing wounds. Under conditions of circadian disruption, such as sleep deprivation, ageing, diabetes and obesity, bone marrow granulopoiesis, peripheral neutrophil ageing, chemokine receptor expression and tissue clearance of neutrophils may become desynchronised. On the one hand, insufficient early neutrophil recruitment can weaken antimicrobial defence, increase bacterial burden and delay wound closure. On the other hand, persistent neutrophil retention or excessive activation can expose the wound to prolonged ROS, NETs and protease activity, trapping the tissue in a state of chronic inflammation and matrix destruction. In diabetes and chronic wounds, excessive neutrophil activation and NET accumulation may contribute substantially to delayed healing (49). Thus, neutrophils are not merely executors of early wound inflammation; their rhythmic recruitment, functional transitions and timely clearance may also influence whether a wound successfully progresses from acute inflammation to tissue repair.

### 3.2 Circadian programming of macrophages and the inflammation-to-repair transition

As central effectors of innate immunity, macrophages are broadly distributed throughout the body and populate diverse organs such as the skin, liver, lungs, spleen, intestine and adipose tissue. They have essential roles in pathogen clearance, inflammatory regulation, tissue repair and the maintenance of homeostasis (50, 51). In contrast to neutrophils, which mainly mediate early wound defence, macrophages have a particularly important role in controlling the duration of inflammation and driving the transition from inflammatory clearance to tissue repair.

After disruption of the skin barrier, macrophages in the wound are derived from two main sources: pre-existing skin-resident macrophages and circulating monocytes. Chemokines such as CCL20 and CCL2, together with growth factors such as PDGF released locally within the injured wound, promote monocyte recruitment to the site of injury, leading to their progressive accumulation within the wound 24–48 hours after damage. During the early phase after injury, wound macrophages are enriched for inflammatory, antimicrobial, phagocytic and debris-clearance programmes. They produce mediators such as TNF, IL-1β and IL-6, amplify local inflammatory responses, and remove pathogens, necrotic tissue and apoptotic neutrophils. As healing progresses, the macrophage compartment becomes increasingly enriched for efferocytic, pro-resolving, angiogenic and matrix-remodelling programmes. These programmes can coexist within individual cells and should not be interpreted as two mutually exclusive macrophage phenotypes. Mediators including IL-10, VEGF, TGFβ and PDGF participate in distinct aspects of inflammatory resolution, angiogenesis, fibroblast regulation and extracellular matrix remodelling (52, 53).

A key determinant of wound healing is whether the inflammatory response can be initiated, constrained and redirected towards repair at the appropriate time. If inflammation persists, the wound microenvironment remains chronically exposed to high levels of pro-inflammatory mediators, proteases and reactive oxygen species, which can impair keratinocyte migration, increase matrix degradation and ultimately lead to chronic non-healing wounds. Conversely, if inflammation terminates prematurely, pathogen and necrotic tissue clearance may be insufficient, increasing the risk of infection. Thus, the central role of macrophages is not merely to participate in inflammation, but to regulate its magnitude and duration and to drive the transition of the wound from a “clearance phase” to a “repair phase”.

The role of macrophages in cutaneous injury repair can therefore be understood as a temporally programmed process of immune regulation (54). Their recruitment, functional-state transitions, phagocytic and efferocytic activities, cytokine secretion and metabolic programmes must all be precisely coordinated within defined temporal windows (55). This suggests that macrophages may be important immune-cell mediators linking circadian rhythms to cutaneous wound healing. Because circadian rhythms can influence immune-cell migration, inflammatory mediator release, metabolic reprogramming and tissue-repair capacity, changes in macrophage-intrinsic rhythmicity may further determine the intensity of post-injury inflammation, the pace of inflammatory resolution and the ultimate quality of wound healing (56).

Research into circadian rhythms in macrophages began in 2007, when Hayashi et al. (33) isolated peritoneal macrophages from mice maintained under 12-hour light and 12-hour dark conditions and demonstrated rhythmic fluctuations in both core clock gene expression and macrophage functions, including phagocytic activity and cytokine release. Keller et al. (57) also reported that more than 8% of the macrophage transcriptome in mice is expressed in a circadian manner. Together, these observations support a substantial role for circadian timing in macrophage function, with Bmal1 emerging as a key molecular regulator. By using a mouse model with myeloid lineage-specific Bmal1 deficiency, Mingyu Huo et al. (58) found that, compared with control mice, these animals developed larger atherosclerotic lesions, had increased numbers of macrophages within lesions, and reported a shift towards a more inflammatory macrophage profile, described in the original study using an M2-like-to-M1-like classification. Kitchen et al. (34) further showed that selective loss of BMAL1 within macrophages enhanced host resistance to pneumococcal pneumonia in mice. Clock-driven circadian rhythms help synchronise the timing of inflammatory responses, phagocytosis and cytokine synthesis in macrophages with the organismal metabolic state, and have been shown to affect the production of inflammatory mediators, including Toll-like receptor 9 (TLR9), TNF and IL-6. In addition, CRY and PER genes have also been implicated in multiple biological processes in macrophages (59, 60).

Circadian regulation of macrophages has also been widely reported in cutaneous wound healing, whereas direct evidence that these states are regulated by circadian clocks within wounds remains limited. Recently, Li Tao et al. (61) found that defective macrophage polarisation leads to impaired wound repair. Amanda et al. (62) similarly reported that macrophages in normal wounds transition towards an M2-like phenotype approximately three days after injury, whereas diabetic wounds are characterised by sustained and dysregulated M1-like macrophage polarisation. Kim et al. found that, under conditions of light–dark cycle disturbance combined with high-fat diet feeding, the experimental group showed increased M1-like macrophage polarisation compared with controls maintained under a normal light–dark cycle (57). Besides, although many of the studies used conventional M1/M2 terminology, this binary framework does not adequately capture the overlapping and context-dependent macrophage programmes present in wounds. We therefore interpret these findings as dynamic shifts amongst inflammatory, phagocytic, efferocytic, pro-resolving, angiogenic and matrix-remodelling states rather than as conversion between two mutually exclusive phenotypes. Skin infection models further support the rhythmic involvement of macrophages in cutaneous injury responses. Leishmania major primarily infects myeloid cells, including neutrophils and macrophages. Silke et al. showed that inoculation with L. major at different times of the circadian cycle significantly altered the magnitude of post-infection inflammation. *In vitro* experiments further demonstrated that parasite adhesion to mouse macrophages exhibits circadian rhythmicity and depends on the host circadian machinery. These findings indicate that macrophage function in the skin injury or infection microenvironment is not a static process, but is regulated by the biological clock. Such rhythmic immune regulation may be an important determinant of cutaneous inflammation, pathogen clearance and tissue-healing outcomes.

### 3.3 Dendritic cells, mast cells and innate lymphoid cells

Dendritic cells, mast cells and innate lymphoid cells, together with neutrophils and macrophages, constitute key cellular components of the skin immune microenvironment. Although evidence for circadian regulation of these cells during wound healing remains relatively limited, existing studies suggest that they may contribute to the temporal control of wound inflammation and repair by regulating antigen presentation, inflammatory amplification, vascular permeability, barrier immunity and tissue-repair responses.

As professional antigen-presenting cells, dendritic cells (DCs) provide a critical link between innate and adaptive immune responses (63, 64). After skin injury or infection, DCs can capture local antigens and present them to naive T cells through MHC-I or MHC-II molecules, thereby initiating subsequent adaptive immune responses. These functions, however, are not constant across the day, but display marked circadian rhythmicity. In mice, the capacity of DCs to enter cutaneous lymphatic vessels and migrate to draining lymph nodes is stronger during the behavioural resting phase, reaching relatively high levels around ZT7–9, and lower during the active phase, around ZT19–21. The circadian pattern of DC migration appears to depend on synchronised molecular clocks within DCs and lymphatic endothelial cells, accompanied by oscillatory regulation of chemotactic and adhesion-associated molecules, including CCR7, CCL21, LYVE-1, CD99 and JAM-A.In addition, loss of the core clock gene BMAL1 leads to persistent mitochondrial fragmentation in DCs, impairing antigen processing and weakening subsequent T-cell activation. Thus, DC rhythmicity may participate in the transition from local wound inflammation to systemic immune activation by influencing antigen transport, immune priming in lymph nodes and T-cell responses.

Mast cells (MCs) are important tissue-resident immune cells in the skin and have traditionally been regarded as the major effector cells of IgE-mediated immediate hypersensitivity reactions. IgE activates mast cells through FcϵRI, an αβγγ tetrameric receptor on the mast-cell surface (65). This activation induces mast-cell degranulation and the release of chemical mediators such as histamine, thereby causing symptoms including sneezing, coughing, pruritus and anaphylactic shock. The link between mast cells and the biological clock was first identified in the mouse thyroid and has subsequently been extensively investigated. The mast-cell-intrinsic clock has an important role in IgE-mediated allergic responses. Studies have shown that cutaneous intradermal responses to histamine, house dust and grass pollen extracts display a pronounced circadian pattern, with allergic responses increasing in the afternoon, peaking in the evening and reaching a trough in the morning (66). Nakamura et al. (35) similarly found that the circadian variation in cutaneous allergic responses was abolished in mice carrying mast-cell-specific mutations in the Clock gene. These findings suggest that the intrinsic mast-cell clock can regulate degranulation capacity and inflammatory mediator release. Although direct evidence that mast-cell rhythms participate in wound healing remains limited, mast cells can influence vasodilation, inflammatory-cell recruitment and tissue remodelling. Their rhythmic activation may therefore represent an important factor regulating the intensity of early wound inflammation and changes in the local microenvironment.

Innate lymphoid cells (ILCs) represent a heterogeneous population of innate lymphocytes characterised by the absence of antigen-specific receptors and the capacity for rapid effector activity. They mainly include natural killer (NK) cells, ILC1s, ILC2s and ILC3s, and are involved in maintaining barrier immune homeostasis, responding to pathogens and commensal microorganisms, shaping adaptive immunity and controlling tissue inflammation (67). As early responders, ILCs can promptly sense and respond to cytokines and danger signals released by tissue-resident cells. ILC1s and NK cells are mainly associated with immunity to intracellular pathogens and antitumour surveillance. ILC2s initiate type 2 immune responses, which are essential for allergic and anti-parasitic immunity. ILC3s are highly enriched in the intestine and mediate antibacterial responses in the gut. Existing evidence indicates that ILCs are also influenced by circadian rhythms. Wang et al. (68) summarised that the functions of intestinal mucosal ILC3s are jointly regulated by environmental rhythmic cues and core clock genes, thereby influencing intestinal barrier homeostasis and mucosal immunity. Fei Teng et al. (69) also found that circadian regulation is essential for ILC3 homeostasis. Cristina et al. (70) showed that clock genes and ILC3-associated transcription factors in intestinal ILC3s exhibit circadian patterns of expression. Moreover, autonomous deletion of Bmal1 in ILC3s leads to disrupted intestinal ILC3 homeostasis, impaired epithelial responsiveness, dysbiosis of the gut microbiota, increased susceptibility to intestinal infection and disturbed lipid metabolism. However, most existing studies have focused on circadian regulation of ILC3s, whereas other ILC subsets remain less well characterised, and reports directly addressing the role of ILCs in skin wound healing are still limited.

Overall, DCs, mast cells and ILCs may participate in the circadian regulation of wound immunity through distinct mechanisms. However, current evidence is largely derived from models of skin inflammation, allergic responses or intestinal mucosal immunity, and direct studies in cutaneous wound healing remain insufficient. Future work should further define the rhythmic distribution and functional states of these cells across different stages of wound repair, as well as their interactions with neutrophils, macrophages and T cells.

### 3.4 Circadian rhythms in adaptive immunity and chronic wound inflammation

The concept of circadian regulation in the adaptive immune system can be traced back to 1976, when Fernandes et al. (71) found that adaptive immune responses in mice exhibited rhythmic variation after sheep red blood cells were administered at different times of day. Fortier et al. (72) subsequently reported rhythmic expression of clock genes in mouse lymph nodes. When T cells isolated from lymph nodes collected across a 24-hour period were stimulated through the T-cell receptor (TCR), T-cell proliferation displayed circadian variation, whereas this proliferation was impaired in mice carrying mutations in clock genes. David et al. (73) also found that lymphocyte trafficking within lymph nodes and lymph displayed a clear circadian pattern: lymphocyte homing to lymph nodes peaked at night, whereas lymphocytes exited tissues during the day.

As central components of adaptive immunity, CD4^+^ and CD8^+^ T cells are involved in the inflammatory and reparative phases of wound healing. CD4^+^ T cells can give rise to distinct helper and regulatory subsets, including Th1, Th2, Th17 and Treg cells, which collectively regulate inflammation, immune tolerance, pathogen elimination and tissue restoration.CD8^+^ T cells mainly mediate cytotoxic responses and can participate in infection control and the maintenance of chronic inflammation (74). Recent evidence further suggests that Treg cells are also subject to circadian regulation. Circadian disruption can alter Treg homeostasis and function, potentially involving clock-controlled pathways such as PER1 expression, although the direct contribution of Treg rhythmicity to cutaneous wound healing remains largely unexplored (75). An autonomous circadian clock operates within T cells and is regulated by canonical clock genes such as Bmal1, Clock, Per1, Per2 and Rev-erbα/β (76). However, the strength and functional significance of circadian rhythmicity differ amongst T-cell subsets. The circadian rhythms of CD4^+^ T cells remain incompletely understood. Bollinger et al. (40) found rhythmic expression of clock genes in human peripheral blood CD4^+^ T cells *in vitro*, with Bmal1, Rev-erbα and Per3 showing the strongest rhythmicity. By contrast, Hemmers et al. (77) reported that molecular clock oscillations in mouse CD4^+^ T cells were of low amplitude, with weak oscillations of Bmal1 and Rev-erbα. Thus, although naive CD4^+^ T cells appear to possess an oscillatory clock mechanism, definitive evidence that this rhythmicity has functional consequences remains limited. Compared with CD4^+^ T cells, CD8^+^ T cells have a more clearly defined intrinsic circadian rhythm, and their functions vary according to circadian time (78, 79). Their antigen responses are stronger during the subjective day, as reflected by greater expansion, increased IFNγ production and enhanced activation of TCR-associated signalling. This rhythm depends on T-cell-intrinsic Bmal1 and enhances responses to vaccination and infectious challenge by promoting early activation pathways, including IRF4 and AKT–mTOR signalling.

At present, studies of B-cell circadian rhythms and their roles in wound healing remain relatively limited. Hemmers et al. found no obvious abnormalities in B cells lacking Bmal1 expression, leading them to propose that even if B cells possess a functional clock mechanism, its contribution to B-cell development is minimal (77). Therefore, compared with T cells, the role of B cells in circadian regulation of wound immunity requires further investigation.

**Figure 2:**
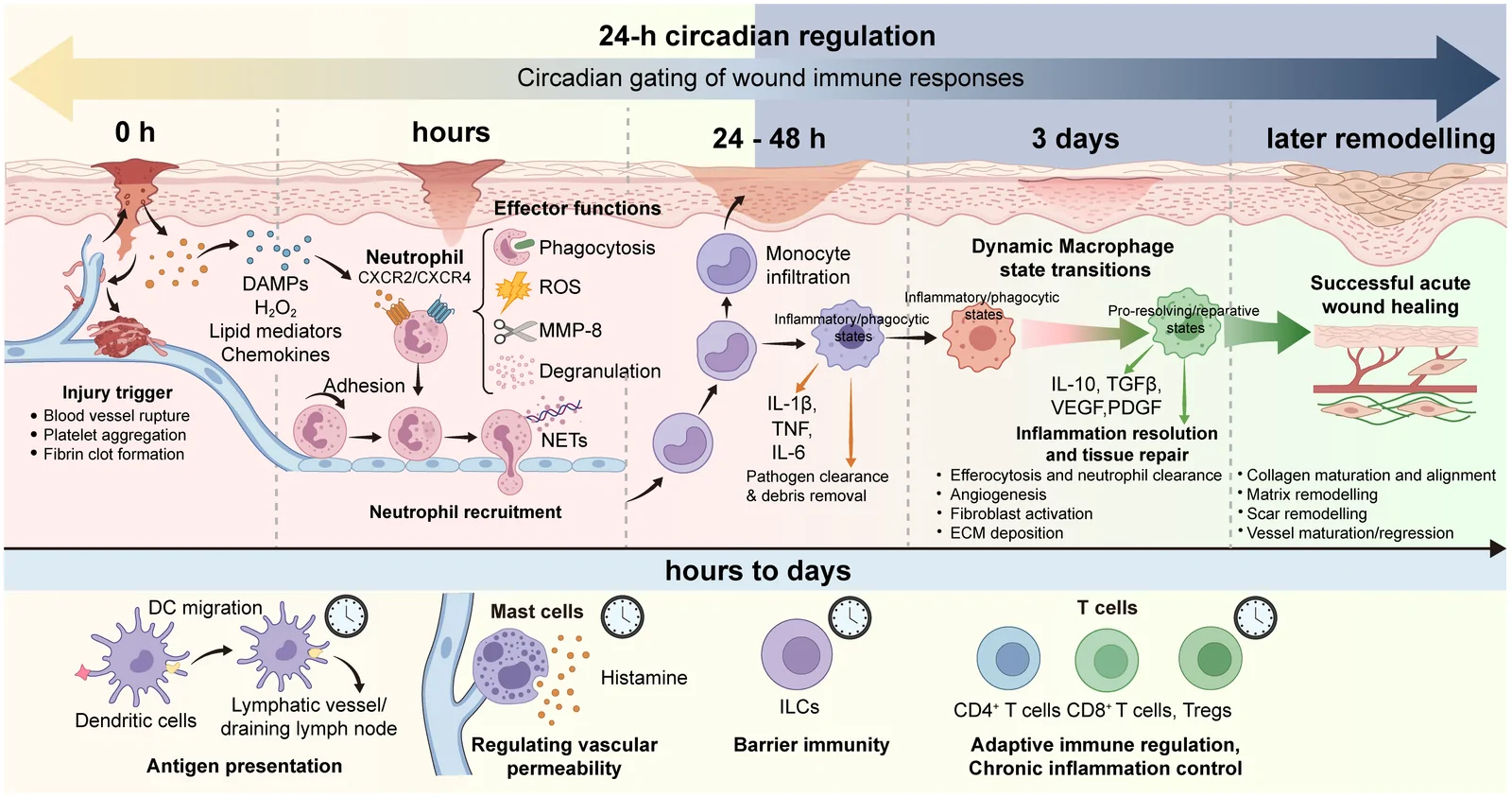
Circadian immune dynamics during cutaneous wound healing. Skin injury initiates haemostasis and releases damage-associated molecular patterns (DAMPs), H_2_O_2_, lipid mediators and chemokines, which promote circadian-gated neutrophil recruitment and activation. Recruited neutrophils contribute to early antimicrobial defence and debris clearance through phagocytosis, reactive oxygen species production, MMP-8 release, degranulation and neutrophil extracellular trap formation. Subsequent monocyte infiltration expands the wound macrophage compartment and supports pathogen clearance and early inflammatory amplification through mediators including IL-1β, TNF and IL-6. As healing progresses, wound macrophages occupy dynamic and overlapping inflammatory, phagocytic, efferocytic, pro-resolving, angiogenic and matrix-remodelling states rather than two mutually exclusive phenotypes. These state changes contribute to efferocytosis, inflammatory resolution, angiogenesis, fibroblast regulation and extracellular matrix remodelling through mediators including IL-10, VEGF, TGFβ and PDGF. During later remodelling, immune resolution supports collagen maturation, scar remodelling and vessel maturation. Other immune populations, including dendritic cells, mast cells, innate lymphoid cells and T cells, act over hours to days to regulate antigen presentation, vascular permeability, barrier immunity and adaptive immune responses.

Immune-cell recruitment and functional transitions during wound healing occur within defined temporal windows, forming a dynamic circadian-regulated immune programme (**Figure 2**).

## 4 Circadian disruption and chronic non-healing wounds

Normal cutaneous wound healing depends on the precise coordination of inflammatory initiation, inflammatory resolution, cell proliferation and tissue remodelling. Circadian rhythms provide an important temporal framework for this process, enabling immune-cell recruitment, inflammatory mediator release, phagocytic clearance, angiogenesis and matrix remodelling to occur within relatively appropriate time windows. However, under conditions such as diabetes, ageing, prolonged bed rest, sleep disturbance and disordered light exposure in hospital environments, both systemic circadian rhythms and local peripheral clocks in the skin may be disrupted (80). In this setting, immune cells may fail to complete recruitment, activation, clearance and functional transitions according to their normal rhythms. Consequently, the wound microenvironment may remain chronically exposed to high levels of inflammatory mediators, proteases, reactive oxygen species and hypoxic signals, causing the wound to become arrested in the inflammatory phase and ultimately develop into a chronic non-healing wound (81). Diabetic wounds are amongst the most representative forms of chronic wounds (82–84).

As a chronic metabolic disease, diabetes mellitus (DM) has emerged as a major worldwide health challenge, partly because it promotes sustained inflammation, compromises angiogenic responses and interferes with the orderly progression of wound repair (83, 84). Patients with diabetes frequently exhibit defective wound healing, which often progresses to chronic non-healing ulcers. These ulcers may develop into severe infections and, in extreme cases, can lead to amputation or death. Diabetic wound repair is complicated by multiple molecular derangements induced by hyperglycaemia, including persistent inflammatory macrophage programmes, impaired acquisition of efferocytic and pro-resolving functions, insufficient angiogenic responses and abnormal extracellular matrix turnover (85). These processes are closely linked to circadian disruption, and a deeper understanding of the molecular basis of this process is necessary to guide the design of more precise therapeutic interventions.

Under physiological conditions, cutaneous wound repair depends on the orderly coordination of haemostasis, inflammation, proliferation and remodelling. This process is not driven solely by injury-derived signals, but is also regulated by the temporal framework provided by circadian rhythms. Neutrophil recruitment, macrophage functional-state transitions, dendritic-cell antigen presentation, mast-cell mediator release, T-cell immunoregulation, angiogenesis and extracellular matrix remodelling must all occur within relatively appropriate time windows to allow the wound to transition from early defence to inflammatory resolution and tissue reconstruction. In diabetes, however, persistent hyperglycaemia can disrupt this precisely coordinated reparative programme, causing wounds to remain in a state of chronic inflammation and inefficient repair. Diabetic wounds are characterised by the persistence of inflammatory macrophage programmes and an inadequate emergence of efferocytic, pro-resolving, angiogenic and tissue-reparative functions (86), thereby contributing to persistent inflammation and altered production of mediators involved in inflammatory resolution, angiogenesis, fibroblast regulation and matrix remodelling, including IL-10, VEGF, TGFβ and PDGF. Because macrophages are themselves regulated by circadian rhythms, disruption of macrophage state dynamics may further impair the transition from inflammation to resolution and repair. At the same time, advanced glycation end products (AGEs) and their receptor RAGE can amplify oxidative stress and inflammatory responses, establishing a self-reinforcing pro-inflammatory microenvironment. Beyond macrophages, persistent neutrophil infiltration and excessive NET formation in diabetic wounds can cause sustained release of proteases, reactive oxygen species and inflammatory mediators, thereby degrading the extracellular matrix and inhibiting angiogenesis and re-epithelialisation. This suggests that circadian disruption may shift neutrophils from early defensive cells into mediators that sustain chronic inflammation. In addition, reduced VEGF release by mast cells, impaired clearance of apoptotic cells by dendritic cells, and decreased numbers and immunosuppressive function of regulatory T cells can all hinder inflammatory resolution and delay the transition from the inflammatory to the proliferative phase. Taken together, diabetic wounds are not merely the consequence of hyperglycaemic injury; rather, they arise from the combined effects of metabolic dysfunction, immune-rhythm imbalance, persistent inflammation, impaired angiogenesis and abnormal matrix remodelling.

## 5 The importance of timing in immunotherapy

As our understanding of circadian regulation of immunity continues to deepen, “when to intervene” has emerged as an important determinant of therapeutic efficacy. The core principle of chronotherapy is not simply to change the treatment modality, but to align pharmacological, surgical or lifestyle interventions with endogenous rhythms, so that therapeutic effects occur during time windows in which immune responses are most sensitive or tissue repair is most active. This concept is particularly relevant to wound healing, because inflammatory initiation, immune-cell recruitment, cytokine release, angiogenesis and matrix remodelling all follow a defined temporal sequence. Any intervention that can be coordinated with these biological processes may, in principle, improve repair efficiency and reduce adverse effects.

Chrono-immunotherapy has shown potential value in multiple diseases (87, 88). For example, the timing of vaccination can influence the formation of immune memory, and morning influenza vaccination induces stronger antibody responses (89). In pharmacological therapy, optimising the timing of drug administration can improve efficacy and reduce side effects without changing the drug itself, thereby complementing conventional drug development. Because melatonin has a short half-life, bedtime administration better aligns with the natural nocturnal peak of melatonin secretion and can shorten sleep-onset latency (90). Beyond drug timing, lifestyle optimisation also represents an application of circadian principles. Maintaining a regular sleep–wake schedule, optimising light exposure and synchronising food intake with the natural light–dark cycle may help realign the biological clock with the external environment and restore systemic clock function (91, 92). Hong Wang et al. (93) found that feeding time can regulate skin function.

In cutaneous wound management, the application of chronotherapy remains exploratory, but it has clear translational potential. First, the timing of debridement, dressing changes, antimicrobial treatment and anti-inflammatory therapy may influence local bacterial clearance, inflammatory mediator release and immune-cell recruitment within the wound. Second, in chronic wounds such as diabetic foot ulcers, pressure injuries and age-related chronic ulcers, circadian-informed care may offer an additional adjunctive dimension to clinical management, although direct evidence supporting formal chronotherapy remains limited. In diabetic foot ulcers, a high risk of obstructive sleep apnoea has been associated with poorer healing, whilst studies of sleep disorders, free-living activity patterns and continuous glucose-monitoring metrics indicate that sleep, rest–activity behaviour and glycaemic variability may provide clinically relevant information for risk stratification and follow-up (94–97). In burns, the time of injury has been associated with subsequent healing kinetics, and burn patients may exhibit persistent alterations in melatonin, cortisol and temperature rhythms; a recent scoping review further highlights the potential relevance of circadian disruption to burn recovery (28, 98, 99). For venous leg ulcers, sleep disruption, fatigue and inflammatory burden have been documented, whereas nocturnal pressure-injury prevention research suggests that sleep-preserving repositioning and care schedules may reduce unnecessary night-time disturbance (100–102). Collectively, these observations support the evaluation of sleep quality, rest–activity patterns, glycaemic variability and nocturnal care burden, as well as supportive measures such as appropriate light exposure, sleep protection and metabolic regularity. However, the available evidence is largely observational or indirect and does not currently justify replacing or rescheduling established interventions according to circadian phase. Prospective studies integrating objective circadian assessment with wound-healing and patient-centred outcomes are required before circadian-informed strategies can be incorporated into routine wound-care pathways. Targeting the mast-cell clock may also represent a valuable chronotherapeutic strategy. Studies have shown that resetting the mast-cell clock has achieved favourable efficacy in patients receiving subcutaneous allergen immunotherapy and in those treated with synthetic glucocorticoids for asthma (103). In summary, these observations indicate that the timing of treatment may influence immune responses, therapeutic efficacy, and the profile of adverse reactions across different clinical settings. These findings are summarised in **Table 1**.

**Table 1:** Representative studies of chronotherapy, timed administration and circadian-informed interventions relevant to cutaneous wound management.

Study	Condition/model and design	Timing or circadian approach	Main findings	Translational relevance and limitations
Hoyle et al. (28)	Fibroblast and keratinocyte models, mouse wounds, and retrospective human burn data	Injury during active/day versus rest/night phases	Circadian actin dynamics altered cell migration; daytime burns healed approximately 60% faster than night-time burns.	Strongest direct evidence that injury timing relates to healing. The human component was observational and did not test treatment scheduling.
Wang et al. (93)	Mouse skin; experimental feeding-time intervention	Time-restricted feeding at different phases	Feeding time shifted the phase of the skin clock and altered UVB-induced DNA-damage responses.	Supports systemic entrainment of the skin clock through feeding schedules; not a wound model and no human outcome data.
Maltese et al. (94)	Diabetic foot ulcer; prospective cohort	Obstructive sleep-apnoea risk as a sleep/circadian-related factor	Higher sleep-apnoea risk was associated with poorer ulcer healing.	Potentially useful for risk stratification. Association does not establish that treating sleep apnoea accelerates healing.
Sheahan et al. (96)	Diabetic foot ulcer; free-living observational study	Objective daily activity monitoring	Patients with active ulcers showed reduced daily activity compared with diabetes control groups.	Supports activity monitoring during follow-up, but daily activity is not equivalent to an endogenous circadian biomarker.
Huang et al. (97)	Postoperative diabetic foot ulcer; clinical cohort with continuous glucose monitoring	Glycaemic time in range	Time in range was independently associated with postoperative outcomes, including the need for secondary surgery.	Supports metabolic monitoring and regularity. Time in range is a glycaemic exposure metric rather than direct evidence of chronotherapy.
Pina et al. (98)	Burn patients; preliminary longitudinal observational study	Serial melatonin, 6-sulfatoxymelatonin, cortisol and temperature profiles	Burn patients exhibited persistent alterations in endocrine and temperature rhythms.	Provides direct human evidence of post-burn circadian disruption; small sample and no circadian intervention was tested.
Winders et al. (100)	Older adults with chronic venous leg ulcers; longitudinal descriptive study during intensive wound care	Sleep, fatigue and inflammatory biomarkers	The study characterised substantial sleep and fatigue burden and examined relationships with TNF-alpha, CRP and IL-6.	Supports incorporating sleep symptoms into assessment; does not establish a causal circadian mechanism or treatment effect.
Kunimitsu et al. (102)	Pressure-injury prevention; scoping review	Nocturnal repositioning and sleep-preserving care schedules	Available studies suggested strategies to balance pressure relief with avoidance of unnecessary sleep disruption.	Clinically relevant to night-time nursing, but evidence was heterogeneous and insufficient to define an optimal schedule.
de Bree et al. (41)	Healthy volunteers and ex vivo monocyte experiments	Morning versus evening BCG vaccination	Morning vaccination induced stronger trained-immunity and adaptive immune responses.	Demonstrates time-dependent immune responsiveness, but the study was not conducted in wound patients.
Bavishi et al. (103)	Subcutaneous allergen immunotherapy; clinical observational study	Injection time of day	Afternoon or evening injections were associated with more frequent local cutaneous reactions than morning injections.	Suggests time of administration may affect treatment tolerability; observational and unrelated to wound healing.
Buttgereit et al. (104)	A multicenter, double-blind, randomised trial involving 288 patients with rheumatoid arthritis	Comparison of bedtime delayed-release prednisone and morning immediate-release prednisone	Delayed-release formulations significantly reduce the duration of morning stiffness, with a similar safety profile.	Direct evidence demonstrates that aligning the peak of drug efficacy with the nocturnal rise in inflammation improves therapeutic outcomes.

**Figure 3:**
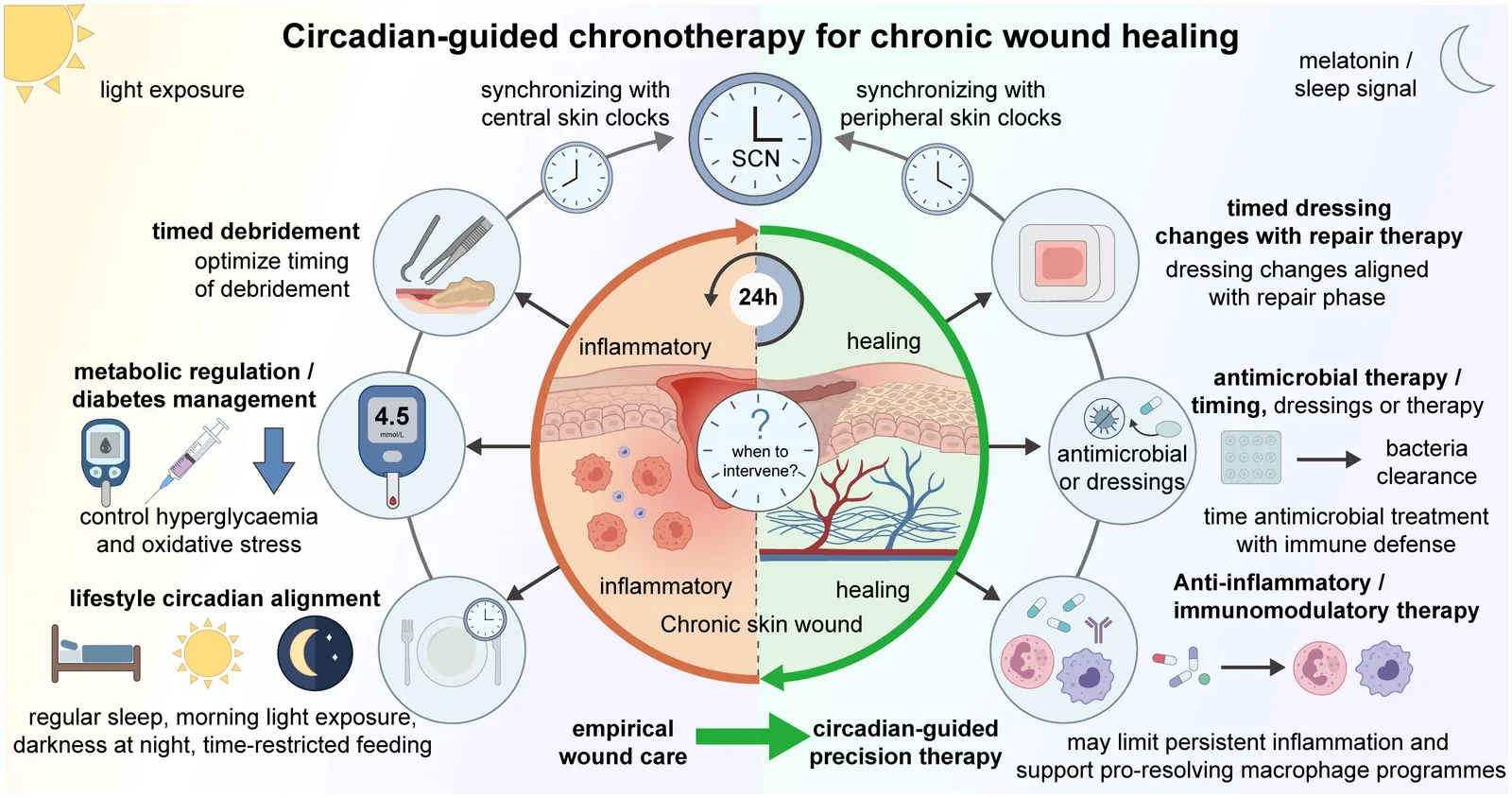
Circadian-guided chronotherapy for chronic wound healing. Circadian-guided chronotherapy aims to align chronic wound care with central and peripheral skin clocks. Timed debridement, dressing changes, antimicrobial therapy, immunomodulation, metabolic control and lifestyle circadian alignment may help reduce persistent inflammation, enhance bacterial clearance and promote tissue repair. This strategy shifts chronic wound management from empirical care towards time-informed precision therapy. Whether scheduling these interventions according to time of day or circadian phase improves wound outcomes remains to be established clinically.

Taken together, direct wound-related evidence is currently dominated by mechanistic studies and observational clinical cohorts, whereas randomised evidence for treatment timing is derived largely from non-wound settings. Accordingly, timed debridement, dressing changes, antimicrobial administration and wound-directed immunomodulation should be regarded as testable translational hypotheses rather than established clinical recommendations. But these findings support the concept that wound-care interventions may be optimised by aligning treatment timing with endogenous circadian rhythms (**Figure 3**).

## 6 Discussion

Cutaneous wound healing is not merely a reparative process sequentially composed of haemostasis, inflammation, proliferation and remodelling; it is also a temporally organised immune programme regulated by circadian rhythms (36). Traditional studies have largely focused on the spatial distribution and functional changes of different cells and molecules within the wound microenvironment, whereas circadian research suggests that wound repair is also governed by a temporal dimension. Immune cells do not participate in wound healing in a static manner; their recruitment, activation, cytokine release, metabolic state and functional transitions may all vary according to circadian timing (99). Thus, the time at which injury occurs may influence the capacity of the host to initiate inflammation and activate tissue repair.

Amongst these immune populations, neutrophils and macrophages may occupy central positions. Amongst the cells discussed in this Review, neutrophils have the best-characterised circadian regulatory machinery. BMAL1–CXCL2 signalling and the CXCR2/CXCR4 axes regulate neutrophil ageing, mobilisation, tissue trafficking and clearance (32, 46–48). Nevertheless, most mechanistic evidence has been obtained from homeostatic, infectious or non-wound inflammatory models, and direct evidence linking disruption of the neutrophil-intrinsic clock to cutaneous wound outcomes remains limited (49). Neutrophils determine the efficiency of early antimicrobial defence and inflammatory initiation in the wound, but if they persist or become excessively activated, they may aggravate tissue damage through ROS, proteases and NETs. Macrophages have the strongest biological relevance to the transition from inflammation to resolution and repair because of their roles in phagocytosis, efferocytosis, cytokine regulation, angiogenesis and matrix remodelling (50, 52–56). Macrophage-intrinsic clocks regulate inflammatory mediator production, motility, phagocytosis and metabolism in several experimental systems (33, 34, 57–60). However, these circadian mechanisms have rarely been directly examined in cutaneous wounds, whereas wound studies have mainly described dynamic macrophage activation states without establishing clock-dependent causality (61, 62). Beyond neutrophils and macrophages, dendritic cells, mast cells, innate lymphoid cells and T cells may also participate in the circadian regulation of wound immunity. By influencing antigen presentation, allergic responses, barrier immunity and adaptive immune responses, these cells collectively shape the local immune microenvironment of the wound (105).

Amongst the core clock components, BMAL1 currently has the strongest mechanistic support. It participates in neutrophil ageing and trafficking, macrophage inflammatory and phagocytic functions, and lymphocyte responses (32, 34, 58, 77). However, its effects are highly cell- and context-dependent. For example, loss of myeloid BMAL1 can aggravate inflammatory pathology in some settings but enhance antimicrobial defence in others (34, 58). BMAL1 should therefore be regarded as a mechanistic hub rather than an immediately actionable therapeutic target. REV-ERBα/NR1D1 may be more amenable to pharmacological modulation because it links circadian timing with inflammatory and metabolic regulation (15, 59), although its wound-specific efficacy and safety remain insufficiently established. PER and CRY proteins also regulate inflammatory signalling (59, 60), but current evidence does not support their prioritisation as wound-healing targets.

In addition to immune cells and classical skin-resident cells, adipocytes within the subcutaneous tissue may represent another potential circadian-regulated component of wound repair. Recent studies have demonstrated that adipocytes contribute to tissue regeneration by promoting fibroblast recruitment and modulating macrophage functions through adipokines and inflammatory mediators. Given that adipose tissue possesses intrinsic circadian clocks that regulate metabolic homeostasis, inflammatory responses and immune cell interactions, circadian disruption may potentially influence adipocyte-mediated regulation of wound healing. However, direct evidence linking adipocyte-intrinsic circadian mechanisms to cutaneous wound repair remains scarce. Future studies integrating adipose tissue biology, circadian profiling and spatial-temporal analyses of wound healing may provide further insights into this potential regulatory axis (106–109).

Circadian disruption associated with diabetes, ageing, obesity, sleep disturbance and abnormal light exposure may contribute to chronic wound pathology by desynchronising immune-cell recruitment, inflammatory resolution and tissue-repair programmes (80–86). Nevertheless, these conditions also independently alter vascular function, metabolism, innervation and host defence. Circadian disruption should therefore be considered one component of a multifactorial pathological network rather than the sole cause of impaired healing.

However, direct evidence for circadian variation in immune cells within human wounds remains limited, and these conclusions require further validation through time-series sampling, single-cell sequencing, spatial omics and prospective clinical studies. Overall, circadian rhythms provide a new theoretical framework for understanding cutaneous wound healing. Incorporating “when it occurs” and “when to intervene” into wound research and treatment may help move the management of chronic non-healing wounds from empirical care towards circadian-guided precision therapy.
